# Supplementary material for: Effects of total intravenous anesthesia on postoperative quality of recovery and the levels of inflammatory factors in patients undergoing retroperitoneal endoscopic surgery in urology: Study protocol for a randomized, controlled trial
Source: PLoS One. 2026 Apr 29;21(4):e0347293. doi: 10.1371/journal.pone.0347293 (PMC13128117; doi:10.1371/journal.pone.0347293)
Supplement: S2 File — (DOCX) [file pone.0347293.s002.docx]

**Effects of total intravenous anesthesia on postoperative quality of recovery and the levels of inflammatory factors in patients undergoing retroperitoneal endoscopic surgery in urology: Study protocol for a randomized, controlled trial**

***Corresponding author:** Dr Wangming Li, Department of Anesthesiology, Lianyungang Hospital of Traditional Chinese Medicine, Lianyungang City, Jiangsu, China. (E-mails: [lwmqxylyg@163.com](mailto:lwmqxylyg@163.com))

**Competing interest:** None declared

**Date and protocol version:** 20 September 2025, Version 1.0

**Introduction**

Compared with transperitoneal laparoscopic approach, retroperitoneoscopic approach has many advantages in urological surgery, such as shorter puncture path, shorter operative time, less blood loss, less postoperative pain, and faster recovery[1-4]. However, restricted to the retroperitoneal anatomy, the limited working space can make the procedure technically challenging, higher insufflation pressures (20–25 mmHg) of CO_2_ is needed to create a larger working space[5, 6]. More CO_2_ is absorbed into the blood, causing harm to the human internal environment.

Propofol-based total intravenous anesthesia (TIVA) and sevoflurane inhalation anesthesia are two general anesthesia techniques commonly used in clinical practice. Compared with inhalation anesthesia, whether TIVA can provide better postoperative quality of recovery is influenced by the type of surgery[7-9]. The Global Quality of Recovery- 40 (QoR-40) questionnaire was specifically designed to measure the health status of patients after surgery and anesthesia, and it involves 5 dimensions: physical comfort, physical independence, emotional state, psychological support and pain[10]. On the strength of its reliability and validity, QoR-40 has a broad clinical application[11, 12] . Peripheral inflammatory factors can lead to inflammatory reactions such as vasodilation, increased permeability, leukocyte exudation, and even increase the permeability of the blood-brain barrier. Inflammatory factors enter the central nervous system, neurons are damaged or die, resulting in brain tissue damage. By measuring and comparing the levels of perioperative inflammatory factors, we can predict the postoperative recovery of patients[13, 14].

Therefore, the objective of our study is to compare the effects of TIVA and inhalation anesthesia on the quality of recovery by using the QoR-40 questionnaire, and the levels of inflammatory factors in patients undergoing urological retroperitoneoscopic surgery.

**Materials and methods**

This is a prospective, parallel, double-blinded, randomized controlled trial, which will be administered at the Guanyun People’s Hospital.

***Eligibility criteria***

***Inclusion criteria***

Patients who are 18-65 years old with American Society of Anesthesiologists (ASA) physical status I-III and scheduled for Urological Retroperitoneoscopic Surgery under general anesthesia will be eligible for inclusion.

***Exclusion criteria***

Patients who meet the following one or more exclusion criteria will be excluded from the study: (1) unplanned or emergency surgery; (2) those with mental illness, cognitive dysfunction, chronic pain disease, long-term use of sedative and analgesic drugs or alcohol abuse; (3) severe liver and kidney disease;(4) severe cardiopulmonary disease; (5) pregnant women and lactating women ;or (6) those who are allergic or contraindicated to the drugs used in the research.

***Withdrawal criteria***

Withdrawal criteria includes: (1) withdrawal of informed consent or request to withdraw from the study during the observation period; (2) subjects or researchers know the experimental grouping rate is more than 20 %; or (3) PaCO2 ≥ 90mmHg and PH ≤7.15 during the operation.

***Study implementation***

***Anesthesia and monitoring methods***

All patients will be fasting for 6-8 h and water deprivation for 2 h before operation, and will not receive any preoperative medication. Baseline blood pressure and heart rate will be obtained in the preoperative preparation area. After entering the operating room, patients will receive a standard monitoring including electrocardiography (ECG) and pulse oximetry (SpO2). An intra-arterial cannula will be placed in the radial artery for continuous arterial pressure measurement. Anesthetic depth will be measured using the Bispectral index. End-tidal carbon dioxide concentration (PetCO2) will be monitored after intubation. The participants will be divided into the total intravenous anesthesia group (group T) or the inhalation anesthesia group (group C) according to the allocation.

***Intravenous anesthesia induction***

All patients will be continuously infused with 0.9% sodium chloride at the rate of 6-8 mL/kg/h, and preoxygenated with 100% oxygen at the rate of 6 L/min through the mask during the induction period .The two groups will receive the same anesthesia induction methods : 1.5–2mg/kg of propofol, if the BIS value is ≤ 60, 2 mg/kg of cisatracurium and 0.5μg/kg of sufentanil will be administered. If the BIS value is *>* 60, 1 mg/kg of propofol will be immediately added, with an interval of *>* 1min between each bolus, until the BIS value is ≤ 60. Volume-controlled ventilation will be performed (tidal volume 6–8 ml/kg, respiratory rate12–18 times/min and PEEP 5 cmH₂O ) after endotracheal intubation.

***Anesthesia maintenance***

Anesthesia maintenance is defined as the time from the end of induction to the cessation of anesthesia (the infusion or inhalation of anesthetic drugs will be stopped immediately after the end of surgery). Group T will receive continuous propofol infusion at a rate of 4–12 mg/kg/h for maintenance, whereas Group C will receive inhalation of sevoflurane at 1.0-3.0% for maintenance. Remifentanil will be administered at a rate of 0.05–0.2 μg/kg/min, cisatracurium and sufentanil will be added in both groups according to requirements of the surgery. The depth of anesthesia will be titrated to BIS values of 40–55 via the manual adjustment of propofol infusion or inhalation of sevoflurane. Upon completion of the surgery, all anesthetic agents will be discontinued. All patients will be provided with patient-controlled intravenous analgesia (PCIA) (1.5 μg/kg of sufentanil + 0.9% normal saline, total volume 100 ml 2 ml/h for 48 h). The self-controlled capacity and the locking time will be 0.5ml and 15 minutes, respectively.

***Recovery of anesthesia***

After tracheal extubation, patients will be transferred to a post-anesthesia care unit (PACU) and receive oxygen supplementation of 3 L/min via a nasal catheter. When the Steward awakening score ≥4 points is reached, patients will be discharged from the PACU to the surgical wards.

***Outcomes***

***Primary outcomes***

The primary outcome of this study is global Quality of Recovery-40 (QoR-40) score on POD 1. The QoR-40 contains 40 items assessing five dimensions of recovery: emotions, physical comfort, psychological support, physical independence, and pain, which scores range from 40 to 200. QoR-40 has been widely used in clinical practice and research for various surgical types and anesthesia techniques.

***Secondary outcomes***

The secondary outcomes include QoR-40 score on POD 2 and POD 3, levels of proinflammatory cytokines (tumor necrosis factor-α, interleukin -1β, and interleukin -6), hemodynamic parameters, arterial and end tidal carbon dioxide pressure, pain scores at rest and while coughing, extubation time , length of PACU stay, ramsay sedation score on awakening, awakening time , adverse events (postoperative nausea and vomiting, headache, respiratory depression ,hypoxemia).

***Sample size estimation***

The primary outcome that will be assessed in this study is the QoR-40 score on postoperative day (POD) 1. Previous studies reported that the minimal clinically important difference (MCID) of QoR-40 score was 6.3 points[10], based on our institutional data, the Standard Deviation was 9.3 points. Based on this assumption, 36 patients in each group would be required with a power of 80% and at an α level of 0.05. To allow for 10% possible dropouts, a total of 80 patients (n = 40 in each group) will be enrolled. The sample size is calculated using the PASS software (version 15.0.5, NCSS, LCC, Kaysville, UT).

***Data collection and registration***

Trained independent investigators who are unaware of group assignment will collect patients’ demographic data and baseline characteristics. The primary and secondary outcome measures as well as other non-outcome perioperative data will be collected. The values of MAP、HR、BIS、ETCO2 and PaCO2 will be recorded at five timepoints: baseline, 30 mins into the surgery, 60 mins into the surgery, the end of surgery, and immediately after tracheal extubation. Postoperative pain will be evaluated using the Numerical Rating Scale, ranging from 0 to 10 (0=no pain, 10=the most severe imaginable pain). Levels of proinflammatory cytokines and NRS will be recorded at postoperative 2,6,24h. All data will be entered into the electronic case report forms(eCRFs) under the supervision of trained research personnel. Investigators will conduct an interim analysis to make an informed decision on whether to continue the trial or modify the study design to optimize the trial results. After the completion of data registration, the electronic database will be locked. After de-identification, the database will be sent to an independent statistician for statistical analysis.
***Statistical analysis***

The demographic data and baseline characteristics will only be expressed using descriptive statistics and will not be compared between groups. According to the data distribution, continuous data will be expressed as means and standard deviations, or median and interquartile ranges. Categorical data will be presented as numbers and percentages. The differences between groups will be analyzed by independent t-test, repeated measures analysis of variance, or Mann Whitney rank sum test, Chi-squared test, or Fisher exact test as appropriate. All data will be analyzed using the SPSS software (version 21.0; IBM SPSS, Chicago, IL) by the independent statistician. A two-sided P value < 0.05 indicates a statistically significant difference.

**Ethical considerations and declarations**

Our research is conducted according to the principles of the Declaration of Helsinki. Written informed consent will be obtained from all participants and/or their legal representatives. The investigator must report all Serious Adverse Events (SAEs) to the sponsor (the corresponding author in the trial) immediately and inform the Ethics Committee accordingly. The organizer is responsible for the continuous safety assessment of anesthesia protocol, if SAE occurs, the test will be terminated, and if SAE occurs, the participants will be compensated.

**Informed consent**

Researchers will obtain signed informed consent from each subject before surgery, and be responsible for explaining the purpose, methods, expected benefits and potential risks of the clinical trial to the subjects. If the new safety study indicates that the benefit risk assessment has changed significantly, the informed consent form will be reviewed and modified, and all study participants will be provided with access to new information. Participants will receive the revised informed consent form, and researchers will obtain their informed consent form to continue the study. The study participants will be screened according to the inclusion and exclusion criteria after signing the informed consent.

**References:**

1. Sasagawa I, Suzuki Y, Itoh K, Izumi T, Miura M, Suzuki H, et al. Posterior retroperitoneoscopic partial adrenalectomy: clinical experience in 47 procedures. Eur Urol. 2003;43(4):381-5. http://doi.org/10.1016/s0302-2838(03)00087-3

2. Constantinides VA, Christakis I, Touska P, Palazzo FF. Systematic review and meta-analysis of retroperitoneoscopic versus laparoscopic adrenalectomy. Brit J Surg. 2012;99(12):1639-48. http://doi.org/10.1002/bjs.8921

3. Conzo G, Tartaglia E, Gambardella C, Esposito D, Sciascia V, Mauriello C, et al. Minimally invasive approach for adrenal lesions: Systematic review of laparoscopic versus retroperitoneoscopic adrenalectomy and assessment of risk factors for complications. Int J Surg. 2016;28 Suppl 1:S118-23. http://doi.org/10.1016/j.ijsu.2015.12.042

4. Feciche BO, Barbos V, Big A, Porav-Hodade D, Cumpanas AA, Latcu SC, et al. Posterior Retroperitoneal Laparoscopic Adrenalectomy: An Anatomical Essay and Surgical Update. Cancers. 2024;16(22). http://doi.org/10.3390/cancers16223841

5. Walz MK, Alesina PF, Wenger FA, Deligiannis A, Szuczik E, Petersenn S, et al. Posterior retroperitoneoscopic adrenalectomy--results of 560 procedures in 520 patients. Surgery. 2006;140(6):943-8, 948-50. http://doi.org/10.1016/j.surg.2006.07.039

6. Schreinemakers JM, Kiela GJ, Valk GD, Vriens MR, Rinkes IH. Retroperitoneal endoscopic adrenalectomy is safe and effective. Brit J Surg. 2010;97(11):1667-72. http://doi.org/10.1002/bjs.7191

7. Joe YE, Kang CM, Lee HM, Kim KJ, Hwang HK, Lee JR. Quality of Recovery of Patients Who Underwent Curative Pancreatectomy: Comparison of Total Intravenous Anesthesia Versus Inhalation Anesthesia Using the QOR-40 Questionnaire. World J Surg. 2021;45(8):2581-90. http://doi.org/10.1007/s00268-021-06117-0

8. Lee WK, Kim MS, Kang SW, Kim S, Lee JR. Type of anaesthesia and patient quality of recovery: a randomized trial comparing propofol-remifentanil total i.v. anaesthesia with desflurane anaesthesia. Brit J Anaesth. 2015;114(4):663-8. http://doi.org/10.1093/bja/aeu405

9. Niu Z, Gao X, Shi Z, Liu T, Wang M, Guo L, et al. Effect of total intravenous anesthesia or inhalation anesthesia on postoperative quality of recovery in patients undergoing total laparoscopic hysterectomy: A randomized controlled trial. J Clin Anesth. 2021;73:110374. http://doi.org/10.1016/j.jclinane.2021.110374

10. Myles PS, Myles DB, Galagher W, Chew C, MacDonald N, Dennis A. Minimal Clinically Important Difference for Three Quality of Recovery Scales. Anesthesiology. 2016;125(1):39-45. http://doi.org/10.1097/ALN.0000000000001158

11. Gornall BF, Myles PS, Smith CL, Burke JA, Leslie K, Pereira MJ, et al. Measurement of quality of recovery using the QoR-40: a quantitative systematic review. Brit J Anaesth. 2013;111(2):161-9. http://doi.org/10.1093/bja/aet014

12. Myles PS, Weitkamp B, Jones K, Melick J, Hensen S. Validity and reliability of a postoperative quality of recovery score: the QoR-40. Brit J Anaesth. 2000;84(1):11-5. http://doi.org/10.1093/oxfordjournals.bja.a013366

13. Lv X, Li X, Guo K, Li T, Yang Y, Lu W, et al. Effects of Systemic Lidocaine on Postoperative Recovery Quality and Immune Function in Patients Undergoing Laparoscopic Radical Gastrectomy. Drug Des Devel Ther. 2021;15:1861-72. http://doi.org/10.2147/DDDT.S299486

14. Wu Y, Chen Z, Yao C, Sun H, Li H, Du X, et al. Effect of systemic lidocaine on postoperative quality of recovery, the gastrointestinal function, inflammatory cytokines of lumbar spinal stenosis surgery: a randomized trial. Sci Rep-Uk. 2023;13(1):17661. <http://doi.org/10.1038/s41598-023-45022-5>

**Appendix A**

| Table 1 Schedule of patient enrolment, study interventions and outcome assessment | | | | | | | | | |
| --- | --- | --- | --- | --- | --- | --- | --- | --- | --- |
|  | Study period |  |  |  |  |  |  |  |  |
|  | Enrolment | Allocation | Post-allocation |  |  |  |  |  |  |
|  |  | 2 hours |  |  |  |  |  |  |  |
|  | Preoperative | before |  |  | POS | POS | POS | POS | POS |
| Time point | visit | surgery | intraoperatively | PACU | 2h | 6h | 24h | 48h | 72h |
| Patient enrolment |  |  |  |  |  |  |  |  |  |
| Eligibility criteria | **×** |  |  |  |  |  |  |  |  |
| Written informed  consent | **×** |  |  |  |  |  |  |  |  |
| Demographic data | **×** |  |  |  |  |  |  |  |  |
| Baseline  characteristics | **×** |  |  |  |  |  |  |  |  |
| Randomisation/ |  | **×** |  |  |  |  |  |  |  |
| allocation |  |  |  |  |  |  |  |  |  |
| Study interventions |  |  |  |  |  |  |  |  |  |
| Total intravenous |  |  | **×** |  |  |  |  |  |  |
| anaesthesia |  |  |  |  |  |  |  |  |  |
| Inhalation |  |  | **×** |  |  |  |  |  |  |
| anesthesia |  |  |  |  |  |  |  |  |  |
| Outcome assessment |  |  |  |  |  |  |  |  |  |
| QoR-40 scores | **×** |  |  |  |  |  | **×** | **×** | **×** |
| Postoperative pain  scores |  |  |  |  | **×** | **×** | **×** |  |  |
| Length of PACU  stay |  |  |  | **×** |  |  |  |  |  |
| Levels of | **×** |  |  |  | **×** | **×** | **×** |  |  |
| proinflammatory |  |  |  |  |  |  |  |  |  |
| cytokines |  |  |  |  |  |  |  |  |  |
| Hemodynamic |  |  | **×** |  |  |  |  |  |  |
| parameters |  |  |  |  |  |  |  |  |  |
| ETCO2 |  |  | **×** |  |  |  |  |  |  |
| PaCO2 |  |  | **×** |  |  |  |  |  |  |
| Awakening time |  |  | **×** |  |  |  |  |  |  |
| Extubation time |  |  | **×** |  |  |  |  |  |  |
| Level of sedation |  |  | **×** |  |  |  |  |  |  |
| PONV |  |  |  |  |  |  | **×** |  |  |
| Headache |  |  |  |  |  |  | **×** |  |  |
| Hypoxemia |  |  |  |  |  |  | **×** |  |  |
| Respiratory depression |  |  |  |  |  |  | **×** |  |  |
| According to SPIRIT statement of defining standard protocol items for clinical trials. | | | | | | | | | |
| QoR-40, 40 items of Quality of Recovery;PACU, post-anaesthesia care unit;  PONV, postoperative nausea and vomiting; POS, postoperative; | | | | | | | | | |
| ETCO2,end-tidal carbon dioxide;PaCO2,partial pressure of arterial carbon dioxide. | | | | | | |  |  |  |
| SPIRIT, Standard Protocol Items: Recommendations for Interventional Trials. | | | | | | | | | |
