# Supplementary material for: Effects of total intravenous anesthesia on postoperative quality of recovery and the levels of inflammatory factors in patients undergoing retroperitoneal endoscopic surgery in urology: Study protocol for a randomized, controlled trial
Source: PLoS One. 2026 Apr 29;21(4):e0347293. doi: 10.1371/journal.pone.0347293 (PMC13128117; doi:10.1371/journal.pone.0347293)
Supplement: S3 File — (DOC) [file pone.0347293.s003.doc]

**研究方案**

**项目名称（中文）：**全凭静脉麻醉对泌尿外科腹膜后腔镜手术患者术后恢复质量与炎症因子及术中二氧化碳分压的影响

**项目名称（英文）：**Effects of total intravenous anesthesia on postoperative quality of recovery, inflammatory factors and intraoperative carbon dioxide partial pressure in patients undergoing retroperitoneal endoscopic surgery in urology

**研究单位**：灌云县人民医院

**研究负责人**：钱龙

**一、研究背景**

与经腹腔镜途径相比，后腹膜镜途径在泌尿外科手术中具有诸多优势，例如穿刺路径更短、手术时间更短、出血量更少、术后疼痛更轻、恢复更快[1-4]。然而受限于后腹膜解剖结构，有限的工作空间使操作技术难度增加，需采用更高二氧化碳充气压力（20-25 mmHg）以扩大操作空间[5,6]。过量二氧化碳被吸收进入血液，对人体内环境造成损害。

丙泊酚静脉全麻（TIVA）与七氟烷吸入麻醉是临床常用的两种全身麻醉方案。相较于吸入麻醉，TIVA能否提供更优的术后恢复质量受手术类型影响[7-9]。全球术后恢复质量量表-40项（QoR-40）是专门设计用于评估患者术后麻醉恢复健康状况的量表，涵盖身体舒适度、身体自主性、情绪状态、心理支持及疼痛五维度[10]。凭借其良好的信效度，QoR-40在临床应用中具有广泛适用性[11,12]。外周炎症因子可引发血管扩张、通透性增加、白细胞渗出等炎症反应，甚至增强血脑屏障通透性。炎症因子侵入中枢神经系统后，神经元受损或死亡导致脑组织损伤。通过测量并比较围手术期炎症因子水平，可预测患者术后恢复情况[13,14]。

因此，本研究旨在通过QoR-40问卷评估TIVA与吸入麻醉对泌尿科后腹膜镜手术患者恢复质量的影响，并比较两组炎症因子水平差异。

**二、研究目的**

本研究探讨全凭静脉麻醉对泌尿外科腹膜后腹腔镜手术术后恢复质量评分与炎症因子及术中二氧化碳的影响。

**三、研究概况**

3.1整体的研究设计和计划

本研究为一项前瞻性、双盲、随机、对照、单中心研究。

3.2 研究人群

3.2.1入选标准

年龄18~65岁，ASA分级I~III级，性别不限，择期行腹膜后腹腔镜的泌尿外科手术。

3.2.2排除标准

①非计划或紧急手术；

②合并精神疾病、认知功能异常、慢性疼痛疾病、长期服用镇静镇痛药物或酒精滥用者；

③严重肝肾功能疾病；

④严重心肺功能疾病；

⑤孕妇、哺乳期妇女；

⑥对研究所用药物有过敏或禁忌症者。

3.2.3中途退出标准

受试者撤回知情同意书则退出研究。受试者或研究者知晓实验分组率超过20%则研究终止。术中若发现PaCO2大于90mmHg,PH值小于7.15则研究终止。

3.3病例数及分组方法

共纳入泌尿外科拟行腹膜后腹腔镜手术患者80例，随机数字表法分为试验组（全凭静脉麻醉组）与对照组（吸入麻醉组），每组40例。因两组麻醉维持方法显著不同，无法对主治麻醉医师设盲，但对患者、术前和术后随访评估者、统计分析人员对分组情况均不知情。

3.3试验药物

丙泊酚乳状注射液，规格200mg/20ml，批号12505262，国药准字号 H20223914,江苏盈科生物制药有限公司；枸橼酸舒芬太尼注射液，规格 50ug/ml，批号 AB50200111,国药准字号 H20054171，宜昌人福药业有限责任公司；注射用瑞芬太尼，规格 1mg，批号 AD5030081,国药准字号 H20030197，宜昌人福药业有限责任公司；苯磺顺阿曲库铵注射液, 规格 10mg/5ml，批号 250704XA,国药准字号 H20183042，江苏恒瑞医药股份有限公司；吸入用七氟烷，规格 120ml，批号 25041031,国药准字号 H20213735，上海恒瑞医药有限公司；盐酸麻黄碱注射液，规格 30mg/1ml，批号 240502,国药准字号 H21022412，东北制药集团沈阳第一制药有限公司；硫酸阿托品注射液，规格 0.5mg/1ml，批号2409041,国药准字号 H12020382，北京京丰制药集团；盐酸乌拉地尔注射液，规格 25mg/5ml，批号DD240706,国药准字号 H120234436，浙江赛默制药有限公司；艾司洛尔注射液，规格 0.1g/10ml，批号2502181L,国药准字号 H20243401，哈尔滨三联药业股份有限公司；氟比洛芬酯注射液，规格 50mg/5ml，批号62410301-1,国药准字号 H20183054，远大医学营养科学（武汉）有限公司；

3.4研究步骤及相关检查

术前一天访视病人，签署麻醉及科研项目的知情同意书，向病人说明相关评分量表的使用方法并进行基本资料收集（年龄、性别、BMI、既往合并症等）。按照随机数字表法，将病人随机分为两组，试验组（全凭静脉麻醉组）与对照组（吸入麻醉组）。手术当天不用术前用药，术前禁食8 h、禁饮4 h。患者入室后吸氧并常规开放外周静脉通路，并开始基础液体输注（平衡液，5 ml/kg/h）。患者入手术室后的标准监测包括：心电图（ECG），脉搏氧饱和度（SpO2），无创血压（NIBP），呼气末二氧化碳（PetCO2），BIS监测麻醉深度，局麻下行桡动脉穿刺置管测量动脉血压。两组采用相同的麻醉诱导方式，丙泊酚1.5~2mg/kg、舒芬太尼0.3µg/kg、顺式阿曲库铵2 mg/kg，待药物起效后，完成气管插管，连接麻醉机。麻醉机调整呼吸参数，潮气量6~8ml/kg,呼吸频率16次/分，PEEP 5mmHg,吸入60%氧气，流量为2l/min。试验组麻醉维持丙泊酚剂量4~12 mg·kg^-1^·h^-1^、瑞芬太尼剂量0.05~0.2µg·kg^-1^·min^-1^ 。对照组麻醉维持七氟烷吸入1.0~3.0%、瑞芬太尼剂量0.05~0.2µg·kg^-1^·min^-1^ 。术中使用脑电双频指数BIS监测麻醉深度，调整丙泊酚剂量或七氟烷吸入浓度维持BIS 40~55之间。手术完成拔管后，转入麻醉后恢复室（Postanesthesia care unit PACU），待完全苏醒、可以吞咽、可以抬头、Steward评分≥4分，护送回病房。两组患者术后均使用静脉自控镇痛泵，给予舒芬太尼1.5µg/kg，生理盐水稀释至100ml，设置输注速度2ml/h、单次按压0.5ml、间隔15min。

术中监测麻醉前（T1）、手术30min（T2）、手术60min（T3）、术毕（T4）、拔管（T5）5个时点患者的心率、平均动脉压（MAP）、BIS、ETCO2、PaCO2。记录术中出现低血压，心动过缓，高血压及血管活性药使用情况。于术前，术后2、6、24 h抽取静脉血5 ml，使用ELISA检测方法测定白细胞介素1β（IL-1β）、白细胞介素6（IL-6）及肿瘤坏死因子-α（TNF-α）。在术后2、6、24h使用NRS进行疼痛评分。在术前，术后第1、2、3天,采用40项恢复质量量表（QoR-40）对患者进行评估，该量表包括身体舒适度、心理支持、情绪状态、自理能力及疼痛控制5个方面，总分为200分，得分越高代表术后恢复质量越佳。记录患者苏醒时Ramsay镇静评分，以及苏醒时间、气管拔管时间、麻醉后监护治疗室（PACU）停留时间，并统计术后24 h内恶心呕吐、头痛、呼吸抑制、低氧血症等不良事件的发生率。

Ramsay镇静评分，可以根据患者临床状态分为6分，具体情况如下：1分，是指焦虑、激动或者不安；2分，是指患者是合作、服从或者安静状态；3分，是指患者入睡，对命令有反应；4分，是指患者入睡，对轻度摇晃或大声音刺激有反应；5分，是指患者入睡，对伤害性刺激，如用力压迫有反应；6分，是指患者入睡，对上述刺激无任何反应。NRS评分通常采用0至10分的线性标度：0分，代表完全无痛；10分，代表能想象的最剧烈疼痛。

3.5 终点指标

主要结局指标：术后第1天的QOR40。

次要结局指标：术后第2、3天的QOR40评分；麻醉前（T1）、手术30min（T2）、手术60min（T3）、术毕（T4）、拔管（T5）5个时点患者的心率、平均动脉压（MAP）、BIS、ETCO2、PaCO2；术前，术后2、6、24 h测定白细胞介素1β（IL-1β）、白细胞介素6（IL-6）及肿瘤坏死因子-α（TNF-α）；术后2、6、24 h的NRS疼痛评分；患者苏醒时Ramsay镇静评分，以及苏醒时间、气管拔管时间、麻醉后监护治疗室（PACU）停留时间；术后24 h内恶心呕吐、头痛、呼吸抑制、低氧血症等不良事件的发生率。

**四、不良事件观察**

术中若发现PaCO2大于90mmHg,PH值小于7.15，则研究终止，调整呼吸频率与潮气量，便于CO2的排出，必要时暂停手术，待PaCO2将至正常后再继续手术。术中如发生低血压（收缩压< 90 mmHg或MAP降低幅度超过基础值30%），则静脉注射麻黄素6mg；如发生严重心动过缓（HR< 45次/min），则静脉注射阿托品0.3mg；若发生高血压（收缩压>140mmHg或MAP升高幅度超过基础值30%）和心动过速（HR>100次/min），在麻醉深度和镇痛充分的情况下，可以静脉注射乌拉地尔5mg或艾司洛尔20mg。若术后发生低氧血症（SpO2＜90%），面罩加压辅助呼吸，行血气分析并对症处理。术后疼痛补救治疗方案：如患者要求或NRS评分大于等于3-4分，则给予氟比诺芬酯（凯纷）50mg静脉滴注，必要时24小时内可重复一次。

**五、统计分析**

5.1样本含量估计

依据Myles PS等 ^[1]^ 研究，QoR-40最小临床重要差异( minimal clinical important difference，MCID) 为6.3分，结合预试验结果，术后24 h试验组QoR-40评分为(167. 0±9.3)分，对照组组QoR-40评分为(161.8±6.4)分，设α = 0.05，1－β = 0. 8( 双侧检验) ，两组样本量1∶1，采用PASS 15. 0 软件，每组需样本量36例，考虑10%脱落率，每组需样本量40例。

5.2研究数据的统计与分析

采用SPSS 23.0软件进行统计学分析。正态分布计量资料以均数±标准差（
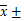
s）表示，组间比较采用两独立样本t检验；非正态分布计量资料以中位数和四分位数间距［*M（IQR）*］ 表示，组间比较采用Mann-Whitney *U*检验。计数资料以例（％）表示，组间比较采用χ^２^ 检验或Fisher精确概率法。*P*＜0.05为差异有统计学意义。

**六、研究相关伦理学**

6.1伦理委员会审核

本方案和书面知情同意书及与受试者直接相关的资料已提交伦理委员会，并获得伦理委员会书面批准。本研究者将向伦理委员会提交研究中期报告。在研究中止和/或完成时，本研究会书面通知伦理委员会；及时向伦理委员会报告所有研究工作中发生的变化（如方案和/或知情同意数的修订），并且在未获得伦理委员会批准之前不得执行这些变动，除非是为了消除对受试者明显且直接的风险而做出的变更。若发生这类情况时，将通知伦理委员会。

6.2知情同意

6.2.1获得知情同意的程序

本研究将向受试者或其法定代理人提供易于理解的并且经伦理委员会批准的知情同意书，并给与受试者或其法定代理人充分的时间考虑本项研究，在从受试者获得签署的书面知情同意书之前，受试者不得入组。 在受试者参与期间，将向受试者提供所有更新版本的知情同意书以及书面信息。知情同意书应作为临床试验的重要文档保留备查。

1. **保密措施**

通过本项目研究的结果可能会在医学杂志上发表，但是我们会按照法律的要求为患者的信息保密，除非应相关法律要求，患者的个人信息不会被泄露。必要时，政府管理部门和医院伦理委员会及其有关人员可以按规定查阅患者的资料。

**八、参考文献**

1. Sasagawa I, Suzuki Y, Itoh K, Izumi T, Miura M, Suzuki H, et al. Posterior retroperitoneoscopic partial adrenalectomy: clinical experience in 47 procedures. Eur Urol. 2003;43(4):381-5. http://doi.org/10.1016/s0302-2838(03)00087-3

2. Constantinides VA, Christakis I, Touska P, Palazzo FF. Systematic review and meta-analysis of retroperitoneoscopic versus laparoscopic adrenalectomy. Brit J Surg. 2012;99(12):1639-48. http://doi.org/10.1002/bjs.8921

3. Conzo G, Tartaglia E, Gambardella C, Esposito D, Sciascia V, Mauriello C, et al. Minimally invasive approach for adrenal lesions: Systematic review of laparoscopic versus retroperitoneoscopic adrenalectomy and assessment of risk factors for complications. Int J Surg. 2016;28 Suppl 1:S118-23. http://doi.org/10.1016/j.ijsu.2015.12.042

4. Feciche BO, Barbos V, Big A, Porav-Hodade D, Cumpanas AA, Latcu SC, et al. Posterior Retroperitoneal Laparoscopic Adrenalectomy: An Anatomical Essay and Surgical Update. Cancers. 2024;16(22). http://doi.org/10.3390/cancers16223841

5. Walz MK, Alesina PF, Wenger FA, Deligiannis A, Szuczik E, Petersenn S, et al. Posterior retroperitoneoscopic adrenalectomy--results of 560 procedures in 520 patients. Surgery. 2006;140(6):943-8, 948-50. http://doi.org/10.1016/j.surg.2006.07.039

6. Schreinemakers JM, Kiela GJ, Valk GD, Vriens MR, Rinkes IH. Retroperitoneal endoscopic adrenalectomy is safe and effective. Brit J Surg. 2010;97(11):1667-72. http://doi.org/10.1002/bjs.7191

7. Joe YE, Kang CM, Lee HM, Kim KJ, Hwang HK, Lee JR. Quality of Recovery of Patients Who Underwent Curative Pancreatectomy: Comparison of Total Intravenous Anesthesia Versus Inhalation Anesthesia Using the QOR-40 Questionnaire. World J Surg. 2021;45(8):2581-90. http://doi.org/10.1007/s00268-021-06117-0

8. Lee WK, Kim MS, Kang SW, Kim S, Lee JR. Type of anaesthesia and patient quality of recovery: a randomized trial comparing propofol-remifentanil total i.v. anaesthesia with desflurane anaesthesia. Brit J Anaesth. 2015;114(4):663-8. http://doi.org/10.1093/bja/aeu405

9. Niu Z, Gao X, Shi Z, Liu T, Wang M, Guo L, et al. Effect of total intravenous anesthesia or inhalation anesthesia on postoperative quality of recovery in patients undergoing total laparoscopic hysterectomy: A randomized controlled trial. J Clin Anesth. 2021;73:110374. http://doi.org/10.1016/j.jclinane.2021.110374

10. Myles PS, Myles DB, Galagher W, Chew C, MacDonald N, Dennis A. Minimal Clinically Important Difference for Three Quality of Recovery Scales. Anesthesiology. 2016;125(1):39-45. http://doi.org/10.1097/ALN.0000000000001158

11. Gornall BF, Myles PS, Smith CL, Burke JA, Leslie K, Pereira MJ, et al. Measurement of quality of recovery using the QoR-40: a quantitative systematic review. Brit J Anaesth. 2013;111(2):161-9. http://doi.org/10.1093/bja/aet014

12. Myles PS, Weitkamp B, Jones K, Melick J, Hensen S. Validity and reliability of a postoperative quality of recovery score: the QoR-40. Brit J Anaesth. 2000;84(1):11-5. http://doi.org/10.1093/oxfordjournals.bja.a013366

13. Lv X, Li X, Guo K, Li T, Yang Y, Lu W, et al. Effects of Systemic Lidocaine on Postoperative Recovery Quality and Immune Function in Patients Undergoing Laparoscopic Radical Gastrectomy. Drug Des Devel Ther. 2021;15:1861-72. http://doi.org/10.2147/DDDT.S299486

14. Wu Y, Chen Z, Yao C, Sun H, Li H, Du X, et al. Effect of systemic lidocaine on postoperative quality of recovery, the gastrointestinal function, inflammatory cytokines of lumbar spinal stenosis surgery: a randomized trial. Sci Rep-Uk. 2023;13(1):17661. <http://doi.org/10.1038/s41598-023-45022-5>
