# Supplementary material for: Effects of total intravenous anesthesia on postoperative quality of recovery and the levels of inflammatory factors in patients undergoing retroperitoneal endoscopic surgery in urology: Study protocol for a randomized, controlled trial
Source: PLoS One. 2026 Apr 29;21(4):e0347293. doi: 10.1371/journal.pone.0347293 (PMC13128117; doi:10.1371/journal.pone.0347293)
Supplement: Attachment — Submitted filename: Response to Reviewers.docx [file pone.0347293.s005.docx]

Response to Reviewers

We greatly appreciate the reviewers for their thoughtful comments and constructive suggestions, which have significantly improved the quality of our manuscript. We have carefully addressed all comments and revised the manuscript accordingly.

Reviewer #1: This is a well-designed and ethically approved randomized controlled trial protocol addressing a relevant clinical question. The primary outcome selection and sample size justification are appropriate. Prior to acceptance, the authors should clarify the hierarchy of outcomes, strengthen the pre-specified statistical analysis plan, and address minor language issues.

Response: We greatly appreciate the reviewer’s positive comments and constructive suggestions on our randomized controlled trial protocol. Following the reviewer’s advice, we have clarified the hierarchy of outcomes in the revised manuscript, strengthened the pre-specified statistical analysis plan, and carefully revised the manuscript to address minor language issues throughout the text. We have clarified these in the revision, “Continuous data will be checked for the normality of the distribution with the **Shapiro-Wilk** test. Data conforming to the normal distribution will be expressed as means with standard deviations and analyzed using an independent samples t-test. Data with the non-normal distribution will be reported as medians with interquartile ranges and examined using the Mann-Whitney rank sum test. Categorical data will be presented as counts, along with the corresponding percentages, and between-group differences will be assessed using the Chi-square test or Fisher's exact test when appropriate. For normally distributed continuous variables measured at multiple time points, a repeated measures analysis of variance will be performed. This will be accompanied by Mauchly's test of sphericity. Greenhouse-Geisser correction will be used when the sphericity assumption is violated. For repeated measures of continuous data that failed to meet normality assumptions, linear mixed-effects models will be constructed to estimate the effects of group, time, and the group-by-time interaction, with random intercepts adjusted for each subject to account for intra-individual correlation. All outcome measures will be analyzed using an intention-to-treat population, encompassing all patients randomized to the respective groups. Missing data will be imputed using the appropriate method, such as averaging the values within the group or using multiple imputation. Multiple comparison corrections for the secondary outcomes will not be performed, so these outcomes should be regarded as exploratory.” (See page 14 lines 284-296, page 15 lines 29-396). We hope these revisions meet the reviewer’s expectations and improve the quality of the manuscript.

Reviewer #2: This protocol describes a single-center, randomized, patient- and assessor-blinded controlled trial investigating whether total intravenous anesthesia (TIVA) improves postoperative recovery compared with inhalation anesthesia in patients undergoing urological retroperitoneoscopic surgery. A total of 80 adult patients (ASA I–III) will be randomized 1:1 to receive either propofol–remifentanil–based TIVA or sevoflurane–remifentanil inhalation anesthesia. The primary outcome is postoperative recovery quality measured by the QoR-40 score on postoperative day 1. Secondary outcomes include QoR-40 scores on days 2 and 3, perioperative inflammatory markers (TNF-α, IL‑1β, IL‑6), pain scores, extubation and awakening times, PACU length of stay, sedation levels, and adverse events. The study aims to clarify whether TIVA enhances recovery quality and attenuates inflammatory responses following retroperitoneoscopic urological surgery.

We sincerely appreciate the reviewer for the careful and constructive comments on our study protocol. We have carefully addressed each concern as detailed below.

1. Sample Size Justification: Explicitly state the statistical test underlying the sample size calculation (e.g., two-sample t-test) and clarify any assumptions regarding normality and equal variances between groups.

Response: We agree with the reviewer’s valuable comment. In the revised manuscript, we have explicitly stated that the sample size calculation was based on a two-sample t-test for the primary outcome (QoR-40 score on postoperative day 1). We have also clarified the assumptions of normality and homogeneity of variances between groups that were applied in the sample size estimation process. The relevant description has been added to the sample size section, “The primary endpoint that will be evaluated in this study is the QoR-40 score on the first postoperative day. Previous studies reported that the minimal clinically important difference (MCID) of the QoR-40 score was 6.3 points[10]. Based on our institutional data, the data were normally distributed and exhibited homogeneity in variance. The standard deviation was 9.3 points, and the sample size was calculated using the two-sample t-test. On the basis of this assumption, it is estimated that 36 patients in each group would be required, with a power of 80% and at an α level of 0.05. To allow for a 10% possible dropout, a total of 80 patients (n = 40 in each group) will be enrolled.” (See page 12 lines 239-245)

2. Handling of Secondary Outcomes and Multiplicity: Multiple secondary outcomes are planned, including repeated QoR‑40 measurements, inflammatory biomarkers, pain scores, and several perioperative endpoints. The protocol does not address whether adjustments for multiple comparisons will be applied or whether secondary outcomes are considered exploratory. Clarification is recommended to avoid inflation of type I error and overinterpretation of secondary findings.

Response: We thank the reviewer for highlighting this important methodological issue. We have now clearly stated in the revised protocol that all secondary outcomes are considered exploratory, and no formal adjustment for multiple comparisons will be performed to avoid overcorrection in this exploratory analysis, “Multiple comparison corrections for the secondary outcomes will not be performed, so these outcomes should be regarded as exploratory.” (See page 15 lines 305-306)

3. Repeated Measures Analysis: QoR‑40 scores and inflammatory markers are measured at multiple postoperative time points. The authors propose repeated-measures ANOVA but do not specify how model assumptions (e.g., sphericity) will be assessed or whether alternative methods (e.g., mixed-effects models) would be considered if assumptions are violated.

Response: We appreciate the reviewer’s professional suggestion. In the revised statistical analysis plan, we have specified that sphericity will be tested using Mauchly’s test for repeated-measures ANOVA. If the sphericity assumption is violated, Greenhouse–Geisser correction will be applied. In addition, we have noted that linear mixed-effects models will be considered as an alternative method for repeated measures data of continuous data that failed to meet normality assumptions, “For normally distributed continuous variables measured at multiple time points, a repeated measures analysis of variance will be performed. This will be accompanied by Mauchly's test of sphericity. Greenhouse-Geisser correction will be used when the sphericity assumption is violated. For repeated measures of continuous data that failed to meet normality assumptions, linear mixed-effects models will be constructed to estimate the effects of group, time, and the group-by-time interaction, with random intercepts adjusted for each subject to account for intra-individual correlation.” (See page 14 lines 292-296, page 15 lines 297-301)

4. Data Management and Storage: Data collection using electronic case report forms (eCRFs), database locking, and de-identification are described. However, the protocol does not specify the data management platform or system used for eCRFs, nor does it describe data security or access controls. Providing this information would strengthen data governance transparency.

Response: We thank the reviewer for this constructive comment. In the revised protocol, we have clearly specified that data collection will be performed using electronic case report forms (eCRFs) established on the Clinical Trial Management Public Platform. We have also supplemented detailed descriptions of data security measures, access control policies, user authorization levels, and database management procedures to enhance transparency and rigor in data governance,“Standard data management procedures will be used to manage all data. This includes double data entry, data validation, query management, and database locking. These procedures will be followed before statistical analysis. To protect participant privacy, de-identification will be conducted by removing all personally identifiable information. Comprehensive data security measures and access control policies will be implemented throughout the study. These include role-based access authorization, encrypted data transmission and storage, regular data backup, and complete audit trails. These measures will ensure data confidentiality, integrity, and traceability.” (See page 13 lines 264-173)

5. Baseline Characteristics: The protocol states that baseline characteristics will be presented descriptively but not statistically compared between groups. This approach is acceptable and consistent with CONSORT recommendations; however, a brief justification for this decision would be helpful for clarity.

Response: We appreciate the reviewer’s suggestion. As noted by the reviewer, not performing statistical comparisons of baseline characteristics is consistent with CONSORT guidelines for randomized controlled trials, because randomization is expected to balance baseline variables, and significance testing of baseline imbalance is generally discouraged. We have added this brief justification in the revised manuscript to clarify the rationale for descriptive presentation only, “According to standard statistical reasoning, testing for demographic and baseline balance is unnecessary and uninformative in randomized controlled trials, because it only evaluates random variation, not clinically significant imbalance.” (See page 14 lines 281-284)

6. Data Summary: Clarify that categorial data will be presented as counts with corresponding percentages.

Response: Thanks for the reviewer’s suggestion. We have revised the data summary section to explicitly state that categorical variables will be summarized as counts, along with the corresponding percentages, “Categorical data will be presented as counts, along with the corresponding percentages, and between-group differences will be assessed using the Chi-square test or Fisher's exact test when appropriate.” (See page 14 lines 289-292)
